# Supplementary material for: Biochemical characterization of the cyclooxygenase enzyme in penaeid shrimp
Source: PLoS One. 2021 Apr 22;16(4):e0250276. doi: 10.1371/journal.pone.0250276 (PMC8062024; doi:10.1371/journal.pone.0250276)
Supplement: S1 Table — (DOCX) [file pone.0250276.s001.docx]

**S1 Table. Analytical characteristics of PGF_2α_ and PGE_2_ using UPLC-HRMS/MS**

| **Compound** | **Linearity range (nM)** | **Linear equation** | **r^2^** | **LOQ**  **(nM)** |
| --- | --- | --- | --- | --- |
| PGF_2α_ | 1.95-125 | y = 2.6415x + 0.0080 | 0.9960 | 1.95 |
| PGE_2_ | 15.6-1000 | y = 4.9329x + 0.1502 | 0.9997 | 15.6 |
