# Supplementary material for: Biochemical characterization of the cyclooxygenase enzyme in penaeid shrimp
Source: PLoS One. 2021 Apr 22;16(4):e0250276. doi: 10.1371/journal.pone.0250276 (PMC8062024; doi:10.1371/journal.pone.0250276)
Supplement: S2 Table — (DOCX) [file pone.0250276.s002.docx]

**S2 Table.** List of prostaglandin biosynthesis genes in identified in *P. monodon* and *P. vannamei*.

| **Gene name** | **Accession number** | **Genomic location** | **Length (amino acids)** | **Closest species** | **Identity** | **E-value** | **Reference** |
| --- | --- | --- | --- | --- | --- | --- | --- |
| ***P. monodon* (Genome assembly NSTDA_Pmon_1 (GCF_015228065.1)** | | | | | | | |
| *Cytosolic phospholipase A2 (cPLA2)* | JN003878 | NC_051399.1 (32101551..32122183) | 998 | *Penaeus vannamei* (XP_027207890) | 95.00% | 0.0 | Rotllant G, et al. Mar Genomics, 2015;  Wimuttisuk W, et al. PLoS One, 2013. |
| *Cyclooxygenase (COX)* | KF501342 | NC_051399.1 (31990440..31997457) | 614 | *Penaeus vannamei* (XP_027218437) | 94.95% | 0.0 | Wimuttisuk W, et al. PLoS One, 2013. |
| *Hematopoietic prostaglandin D synthase* | JN003879 | N/A | 203 | *Chionoecetes opilio* (*KAG0727561)* | 51.72% | 1e-72 | Wimuttisuk W, et al. PLoS One, 2013. |
| *Glutathione-dependent prostaglandin D synthase* | JN003880 | N/A | 153 | *Trinorchestia longiramus* (KAF2369025) | 71.05% | 2e-74 | Wimuttisuk W, et al. PLoS One, 2013. |
| *Microsomal prostaglandin E synthase (PGES1)* | JN003882 | N/A | 145 | *Penaeus vannamei* (XP_027230970) | 91.72% | 5e-85 | Wimuttisuk W, et al. PLoS One, 2013. |
| *Prostaglandin E synthase 2 (PGES2)* | JN003883 | NC_051400.1 (27828101..27869981, complement) | 415 | *Penaeus vannamei* (XP_027219753) | 96.63% | 0.0 | Wimuttisuk W, et al. PLoS One, 2013. |
| *Prostaglandin E synthase 3* | JN003881 | NC_051401.1 (17950954..17963912, complement) | 164 | *Armadillidium vulgare* (RXG70052) | 71.43% | 9e-62 | Wimuttisuk W, et al. PLoS One, 2013. |
| *Prostaglandin F synthase* | JN003884 | N/A | 317 | *Penaeus vannamei* (ROT71128) | 80.91% | 1e-166 | Wimuttisuk W, et al. PLoS One, 2013. |
| *Prostaglandin reductase 1* | XM_037932202 | NC_051402.1 (34195462..34204099) | 338 | *Penaeus vannamei* (XP_027220265) | 95.15% | 0.0 | Prasertlux S, *et al.* Aquaculture, 2011 |
| ***P. vannamei*** | | | | | | | |
| *Cytosolic phospholipase A2 isoform X1 (cPLA2)* | XP_027207888 | NW_020872826.1 (47159..70857) | 1051 | *Penaeus monodon* (AFJ11391) | 94.22% | 0.0 | N/A |
| *Cytosolic phospholipase A2 isoform X2 (cPLA2)* | XP_027207890 | NW_020872826.1 (47159..70857) | 999 | *Penaeus monodon* (AFJ11391) | 94.80% | 0.0 | N/A |
| *Cytosolic phospholipase A2 isoform X3 (cPLA2)* | XP_027207891 | NW_020872826.1 (47159..70857) | 919 | *Penaeus monodon* (AFJ11391) | 95.10% | 0.0 | N/A |
| *Cyclooxygenase (COX)* | XP_027218437 | NW_020869580.1 (231200..238467) | 613 | *Penaeus monodon* (AHA44500) | 94.52% | 0.0 | N/A |
| *Hematopoietic prostaglandin D synthase* | XP_027217035 | NW_020869435.1 (103658..106445) | 194 | *Penaeus monodon* (XP_037795305) | 82.38% | 1e-108 | N/A |
| *Microsomal glutathione S-transferase 1-like* | XP_027230970 | N/A | 144 | *Penaeus monodon* (AFJ11395) | 91.72% | 5E-93 | N/A |
| *Prostaglandin E synthase 2-like* | XP_027219753 | N/A | 415 | *Penaeus monodon* (XP_037785979) | 96.63% | 0.0 | N/A |
| *Prostaglandin E synthase 3* | XM_027354303 | N/A | 164 | *Penaeus monodon* (XP_037786909) | 95.52% | 1e-82 | N/A |
| *Prostaglandin F synthase* | ROT71128 | N/A | 609 | *Penaeus monodon* (AFJ11397) | 80.91% | 2e-166 | N/A |
| *Prostaglandin reductase 1* | XP_027220265 | N/A | 338 | *Penaeus monodon* (XP_037788130) | 94.97% | 0.0 | N/A |

N/A indicates that the information was not available.
