# Supplementary material for: Biochemical characterization of the cyclooxygenase enzyme in penaeid shrimp
Source: PLoS One. 2021 Apr 22;16(4):e0250276. doi: 10.1371/journal.pone.0250276 (PMC8062024; doi:10.1371/journal.pone.0250276)
Supplement: S3 Table — (DOCX) [file pone.0250276.s003.docx]

**S3 Table. Accession number of PmCOX and COX homologs used in the construction of phylogenetic tree.**

| **Organism** | **Species** | **COX isoform** | **GenBank Accesson No.** |
| --- | --- | --- | --- |
| Human | *Homo sapiens* | *COX1* | AY449688.1 |
| Human | *Homo sapiens* | *COX2* | AY382629.1 |
| Sheep | *Ovis aries* | *COX1* | NP_001009476.1 |
| Sheep | *Ovis aries* | *COX2* | NP_001009432.1 |
| Rabbit | *Oryctolagus cuniculus* | *COX1* | NP_001076150.1 |
| Rabbit | *Oryctolagus cuniculus* | *COX2* | NP_001075857.1 |
| Mouse | Mus musculus | *COX1* | NP_032995.1 |
| Mouse | Mus musculus | *COX2* | NP_035328.2 |
| Norway rat | *Rattus norvegicus* | *COX1* | NP_058739.4 |
| Norway rat | *Rattus norvegicus* | *COX2* | NP_058928.3 |
| Fresh water flea | *Daphnia pulex* | *COX* | EFX85708.1 |
| Amphipod | *Gammarus* sp. | *COX* | GQ180796.1 |
| Amphipod | *Caprella* sp. | *COX* | GQ180795.1 |
| Hawaiian volcano shrimp | *Halocaridina rubra* | *COX* | ALG96666.1 |
| American lobster | *Homarus americanus* | *COX* | KM437916.1 |
| Blue crab | *Callinectes sapidus* | *COX* | KM437921.2 |
| Kuruma shrimp | *Penaeus japonicus* | *COX* | LC114985.1 |
| Black tiger shrimp | *Penaeus monodon* | *COX* | KF501342.1 |
| Pacific white shrimp | *Penaeus vannamei* | *COX* | XM_027362636.1 |
