# Supplementary material for: Biochemical characterization of the cyclooxygenase enzyme in penaeid shrimp
Source: PLoS One. 2021 Apr 22;16(4):e0250276. doi: 10.1371/journal.pone.0250276 (PMC8062024; doi:10.1371/journal.pone.0250276)
Supplement: S1 Fig — (DOCX) [file pone.0250276.s004.docx]

**S1 Fig. Multiple sequence alignment of vertebrate COX1 and invertebrate COXs**

*P.monodon* MSTSVLKTMATTGQRGDKGGGRVMFGVAGVILAASFLFMRTPVHETPAPT--PTAAVIDY 58

*P.vannamei* -MSTSVKSMATTGQRGDKGGGRVVFGVAGVILAASFLFMRTPVHETPAPT--PTAAVIDY 57

*M.japonicus* --------MATTGQRGDKGGGRVMFGVAGVILAASFLFMRTPVHETPAPA--PTEAVIDY 50

*C.sapidus* ---------MDKEGREEKGGRRVMVGVAGVVLAASFLFMRGPVQETPVP---STQPTYDY 48

*H.americanus* ----MVKNMDTEQQQEDKGGGRVLFGVAGVVLAASFLFMRGPAQEAPA--APPSPAPLDY 54

*H.rubra* MSTNEVKSMNTLDWFRDDGDKKLVVGVAGIILAASFLFIRSPVTEPATTVIPQATATYDY 60

*Caprella* --------MQAMSDRRR---AIGFIAV--VAVVWSLSFSGKP-------QNNRVATTLDY 40

*Gammarus* --------MDTMNDRRR---TAVLIVI--AALAWNYLGKEKS-------SSIRIATAVDY 40

*O.cuniculus1* -----------MSRSS----PSLRLPV--LLLLLLLLLLPPP----PPVLPADPGAPAPV 39

*O.aries1* -----------MSRQS----ISLRFPL--LL------LLLSP----SPVFSADPGAPAPV 33

*H.sapiens1* ------------MSRS----LLLRFLL--FL------LLLPP----LPVLLADPGAPTPV 32

*R.norvegicus1* -----------MSRRS----LSLQFPL--LLL----LLLLPP----PPVLLTDAGVPSPV 35

*M.musculus1* -----------MSRRS----LSLWFPL--LLL----LLLPPT----PSVLLADPGVPSPV 35

. :

**N79 N93**

*P.monodon* DPCCAYPCENQGICMSKPDR**NYT**CDCTGTGHYGTNCEIPTWSTAIKNKLKPDPEWLHTFI 118

*P.vannamei* DPCCAYPCENQGICMSKPDR**NYT**CDCTGTGYYGTNCEIPTWSTAIKTKLKPDPEWLHTFI 117

*M.japonicus* DPCCAYPCENQGICLSEANR**NYT**CDCTGTGYYGSNCEIPTWSTMIKTKLKPDPEWLHTFL 110

*C.sapidus* DPCCSFPCENQGICMTLQNNSYTCDCTGTAHYGSNCEIPTWGSWISKHLKPDPETIHNLI 108

*H.americanus* DPCCAYPCLNLGVCLSFPSN**NYT**CDCTGTDHYGEHCEIPTWRGWLKKTLKPDPETIHNLI 114

*H.rubra* DPCCGYPCLNQGVCMTWPGN**NYT**CDCTGTGHYGTHCEIPTWSAWLVKKIKPDPESLHNFL 120

*Caprella* DPCCGYPCGNQGVCMSFPDRSYECDCTNTGHYGA**NCS**KAEFMTTVVKLIKPDPEYLHILL 100

*Gammarus* DPCCEFPCQNQGVCMSYADKSYSCDCTNTGYYGV**NCT**SATVMTAVINAVKPDPEYLHTIL 100

*O.cuniculus1* NPCCYFPCQHQGVCVRVALDRYQCDCTRTGYSGP**NCT**VPDLWTWLRSSLRPSPTFVHYLL 99

*O.aries1* NPCCYYPCQHQGICVRFGLDRYQCDCTRTGYSGP**NCT**IPEIWTWLRTTLRPSPSFIHFLL 93

*H.sapiens1* NPCCYYPCQHQGICVRFGLDRYQCDCTRTGYSGP**NCT**IPGLWTWLRNSLRPSPSFTHFLL 92

*R.norvegicus1* NPCCYYPCQNQGVCVRFGLDHYQCDCTRTGYSGP**NCT**IPEIWTWLRSSLRPSPSFTHFLL 95

*M.musculus1* NPCCYYPCQNQGVCVRFGLDNYQCDCTRTGYSGP**NCT**IPEIWTWLRNSLRPSPSFTHFLL 95

:*** :** : *:*: * **** * : * :* : . ::*.* * ::

**N129 R146 N170**

*P.monodon* TSNGWLWAILNRLPFIHKRLMTYVYLSRGDLVDSPPTYESEHSYITLNAYY**NES**FYGRAL 178

*P.vannamei* TSHSWLWAIINRIPFIHKRLMTYVYLSRGDLVDSPPTYESEHSYITLNAYY**NES**FYARTL 177

*M.japonicus* TSNSWLWAILNRIPFIHKRLMTYVYLSRGDMVDSPPTYESDHSYITLNAYF**NES**YYARTL 170

*C.sapidus* TSCWPLWAVINRIPFLHSRIMTYVYLSRGDLVDSPPTYESDHHFITLDAYY**NES**YYGRAL 168

*H.americanus* TSNSLLWAVLNRISFIHKRLMTYVYTSRGDLVDSPPTYESDHTYITLNAYF**NTS**YYGRAL 174

*H.rubra* TSYGWLWAILNRIPFIHDKLMAYVYLSRGDMVDSPPTFESDHSYITLNAYF**NES**YYARAL 180

*Caprella* TSDLWVWKIINNIGFLQDAAMKYIFLSRGDQVDSPVRFESDHSYITLDAYY**NET**YYARTL 160

*Gammarus* TTDLWIWKVINNIPFIHDAAMKYIFLSRGDQVDSPVRFESDHPYTTLDAYF**NET**YFARTL 160

*O.cuniculus1* THVRWFWEFV**N**-**AT**FIRDTLMRLVLTVRSNLIPSPPTYNLDYDYISWEAFS**NVS**YYTRVL 158

*O.aries1* THGRWLWDFV**N**-**AT**FIRDTLMRLVLTVRSNLIPSPPTYNIAHDYISWESFS**NVS**YYTRIL 152

*H.sapiens1* THGRWFWEFV**N**-**AT**FIREMLMRLVLTVRSNLIPSPPTYNSAHDYISWESFS**NVS**YYTRIL 151

*R.norvegicus1* THGYWIWEFV**N**-**AT**FIREVLMRLVLTVRSNLIPSPPTYNTAHDYISWESFS**NVS**YYTRIL 154

*M.musculus1* THGYWLWEFV**N**-**AT**FIREVLMRLVLTVRSNLIPSPPTYNSAHDYISWESFS**NVS**YYTRIL 154

* .* .:* *::. * : *.: : ** :: : : : ::: * ::: * *

**H233**

*P.monodon* PPVPDHCPTPMGVKGPKEYPDVDELVKKVFLRREFIPEPHETNVLFQYYAQHFTHQFFRT 238

*P.vannamei* PPVPEHCPTPMGVKGPKEYPDVDELIKKVFLRREFIPEPHDTNVLFQYYAQHFTHQFFRT 237

*M.japonicus* PPVPEHCPTPMGVKGPKEYPDVDELIKKVFMRREFIPEPHDTNVLFQYYAQHFTHQFFRT 230

*C.sapidus* PPVPAHCPTPMGVAGPKEFPDVDELIKKVFMRREFIPDPHNTNVLFQYYAQHFTHQFFRT 228

*H.americanus* PPVPEHCPTPFGVKGHKDYPDVDMLIKKVFLRSKFLPEPHNSNLLFQYYAQHFTHQFFRT 234

*H.rubra* PPVPEHCPTPLGDKGYKDYPDVDELIKKVFMRREFIPEPHGTNVLFQYYAQHFTHQFFRT 240

*Caprella* PPIPEHCPTPMGVKGVKELPNLDLLMKKVFARKEFIPDPHDTNLLFQYYAQHFTHQFFRT 220

*Gammarus* PPVPTHCPTPMGVAGKKELPDLDMLIQKVFVRRQFLPEPHDTNLLFQYYAQHFTHQFFRT 220

*O.cuniculus1* PSVPKDCPTPMGTKGKKQLPDAQVLAHRFLLRRTFIPDPQGTNLMFAFFAQHFTHQFFKT 218

*O.aries1* PSVPRDCPTPMDTKGKKQLPDAEFLSRRFLLRRKFIPDPQSTNLMFAFFAQHFTHQFFKT 212

*H.sapiens1* PSVPKDCPTPMGTKGKKQLPDAQLLARRFLLRRKFIPDPQGTNLMFAFFAQHFTHQFFKT 211

*R.norvegicus1* PSVPKDCPTPMGTKGKKQLPDIHLLAQRLLLRREFIPAPQGTNVLFAFFAQHFTHQFFKT 214

*M.musculus1* PSVPKDCPTPMGTKGKKQLPDVQLLAQQLLLRREFIPAPQGTNILFAFFAQHFTHQFFKT 214

* :* .****:. * *: *: . * ::.: * *:* *: :*::* ::*********:*

*P.monodon* DYKKGPHLTKGTG-GVDVSNIYGPTEQDRRALRSGVDGKLRTQKINGEDFPPYLKDVPGI 297

*P.vannamei* DYKKGPHLTKGTG-GVDVSNIYGLTEQDRRALRSGVDGKLRTQKINGEDFPPYLKDVPGI 296

*M.japonicus* DYKKGPQLTKGTG-GVDVSNIYGLTEQDRRALRSGVDGKLRTQKINGEDFPPYLKDVPGI 289

*C.sapidus* DYAKGPHLTKGNG-GVDVSNIYGLNEQDRRALRSWENGKLRTQVINGEEFPPYLKDVPAI 287

*H.americanus* DYKKGPHITKGTD-GVDVSNIYGLTEQDRQALRSGVNGKLRTQLINGEEFPPYLKDVPAI 293

*H.rubra* DYKKGPQLTKGTG-GVDVSNIYGLTETDRQALRSRVNGKLRTQVINGEEFPPYLKDVPGY 299

*Caprella* NYTMCPQFTKGNG-GVDVSNIYGLTEQHRRAIRMNSDGKLKYQVINDEHYPPYLRDVQGI 279

*Gammarus*  NYTKGPQFTKGNG-GVDVSNIYGLTERQRRALRSNVDGKLKFQIINGEHFPPYLKDVPGI 279

*O.cuniculus1* SGKMGPGFTKALGHGVDLGHIYGDSLERQYHLRLFKDGKLKYQVLDGEVYPPSVEEAP-V 277

*O.aries1* SGKMGPGFTKALGHGVDLGHIYGDNLERQYQLRLFKDGKLKYQMLNGEVYPPSVEEAP-V 271

*H.sapiens1* SGKMGPGFTKALGHGVDLGHIYGDNLERQYQLRLFKDGKLKYQVLDGEMYPPSVEEAP-V 270

*R.norvegicus1* SGKMGPGFTKALGHGVDLGHIYGDSLERQYHLRLFKDGKLKYQVLDGEVYPPSVEQAS-V 273

*M.musculus1* SGKMGPGFTKALGHGVDLGHIYGDNLERQYHLRLFKDGKLKYQVLDGEVYPPSVEQAS-V 273

. * :**. . ***:.:*** . : :* :***: * ::.* :** :.:.

*P.monodon* SMDYPPHIPIPEEGKFALGHPFFALLPGLFAYATIWVREHNRVCDELVKVHPDWDDERIY 357

*P.vannamei* SMDYPPHIPIPEEGKFALGHPFFALLPGLFAYATIWVREHNRVCDELVKVHPDWDDERIY 356

*M.japonicus* SMDYPPHIPIPENGKFALGHPFFALLPGLFAYATIWVREHNRVCDELVKLHPSWDDERIY 349

*C.sapidus* SMDYPPHVPIPETGKFALGHPFFALLPGLFAYSTIWMREHNRVCDELLKIHPHWDDERLY 347

*H.americanus* TMDYPPHIPIPEHSKFALGHPFFALLPGLFAYATIWVREHNRVCDELQKIHTDWDDERLY 353

*H.rubra* SMDYPANVPIPETGKFALGHPFFALLPGLFAFSTIWVREHNRICDELLKVHPDWSDERLY 359

*Caprella* EMDYPPHIPITEDNKFALGHPFFALLPGLFVFSTIWMREHNRVCDVLKNQHPDWDDERLY 339

*Gammarus* SMEYPPHLPITEDNKFALGHPFFALLPGLFVYSTIWMREHNRVCEVLKEQHPHWDDERLY 339

*O.cuniculus1* LMHYPRGVP--PRSQMAVGQEVFGLLPGLMLYATLWLREHNRVCDLLKAEHPTWDDEQLF 335

*O.aries1* LMHYPRGIP--PQSQMAVGQEVFGLLPGLMLYATIWLREHNRVCDLLKAEHPTWGDEQLF 329

*H.sapiens1* LMHYPRGIP--PQSQMAVGQEVFGLLPGLMLYATLWLREHNRVCDLLKAEHPTWGDEQLF 328

*R.norvegicus1* LMRYPPGVP--PEKQMAVGQEVFGLLPGLMLFSTIWLREHNRVCDLLKEEHPTWDDEQLF 331

*M.musculus1* LMRYPPGVP--PERQMAVGQEVFGLLPGLMLFSTIWLREHNRVCDLLKEEHPTWDDEQLF 331

* ** :* ::*:*: .*.*****: ::*:*:*****:*: * * *.**:::

**Y383 Y413/H414 H416**

*P.monodon* QTARLIITGEVIKITIEDYVQHLSQYKLKLNFEPELTHGTRFQYHNRIHAEFNHLYHWHP 417

*P.vannamei* QTARLIIIGEVIKITIEDYVQHLSQYNLRLTFEPELTHGTRFQYHNRIHAEFNHLYHWHP 416

*M.japonicus* HTARLIIVGEVIKITIEDYVQHLSQYKLKLTFEPELTHGTRFQYHNRIHAEFNHLYHWHP 409

*C.sapidus* QTARLIITGEVIKITIEDYVQHLSQYRLRLTFEPHLTHGTHFQYHNRIHAEFNHLYHWHP 407

*H.americanus* QTARLIITGEVIKITIEDYVQHLSQYKLRLTFEPELTHGTNFQYHNRIHAEFNHLYHWHP 413

*H.rubra* HTARLIVVGEVIKITIEDYVQHLSQYKLRLTFEPELTHGTRFQYHNRIHAEFNHLYHWHP 419

*Caprella* QTAKLIITGEVIKITIEDYVQHLSQYKVDLKFKPQVVHGTRFQFDNRINAEFNHLYHWHP 399

*Gammarus*  HTAKLIITGEVIKITIEDYVQHLSQYKVDLKFKPQVVHGTRFQFHNRINVEFDHLYHWHP 399

*O.cuniculus1* QTTRLILIGETIKIVIEEYVQQLSGYFLQLKFDPEMLFSVQFQYRNRIAMEFNHLYHWHP 395

*O.aries1* QTARLILIGETIKIVIEEYVQQLSGYFLQLKFDPELLFGAQFQYRNRIAMEFNQLYHWHP 389

*H.sapiens1* QTTRLILIGETIKIVIEEYVQQLSGYFLQLKFDPELLFGVQFQYRNRIAMEFNHLYHWHP 388

*R.norvegicus1* QTTRLILIGETIKIIIEEYVQHLSGYFLQLKFDPELLFRAQFQYRNRIALEFNHLYHWHP 391

*M.musculus1* QTTRLILIGETIKIVIEEYVQHLSGYFLQLKFDPELLFRAQFQYRNRIAMEFNHLYHWHP 391

:*::**: **.*** **:***:** * : *.*.*.: . ..**: *** **::******

**N424**

*P.monodon* LIPDTL**NVS**GTDYAIMDMAYSTAPVFKHGLDEFIHSMVRSRAGALT-SRNHAHALYPVLK 476

*P.vannamei* LIPDTL**NVS**GTDYAIMDMAYSTAPVFKHGLDEFIHSMVNSRAGALT-SRNHAHALYPVLK 475

*M.japonicus* LIPDSL**NVS**GTDYAIMDMAYSTAPVFKHGLDEFIHAMVNSRAGALT-NRNHAQVLYPVLK 468

*C.sapidus* LIPDGLEVSNTSYALMDMAFSTAPIFKHGLDNFIHAMVNSRAGALT-ARNHAHVLYPVLK 466

*H.americanus* LIPDTVKVNDTEYAIMDMAFSTAPVFKHGLDNFIHAMATNRAGALT-TRNHAHTLYPVLK 472

*H.rubra* LIPDALEVNGTSYAIMDMAYSTAPIFKHGLDEFIHSMVKSRAGALT-NRNHANVLYPILK 478

*Caprella*  LIPDGIQVEDKYYSLMDMAFSTKSVFTHGLDKFIESMATSRAGKLS-HSNHPLVTLPVLK 458

*Gammarus* LIPEGIKVEDSYYSLMDMAFSTKSVFTHGLDAFVKALVTNRAGKLT-SRNHSPVTVPVLK 458

*O.cuniculus1* LMPDSFQVGSQEYSYEQFLF**NTS**MLVDYGVEALVDAFSRQSAGRIGGGRNIDHHVLHVAV 455

*O.aries1* LMPDSFRVGPQDYSYEQFLF**NTS**MLVDYGVEALVDAFSRQPAGRIGGGRNIDHHILHVAV 449

*H.sapiens1* LMPDSFKVGSQEYSYEQFLF**NTS**MLVDYGVEALVDAFSRQIAGRIGGGRNMDHHILHVAV 448

*R.norvegicus1* LMPDSFQVGSQEYSYEQFLF**NTS**MLVDYGVEALVDAFSRQRAGRIGGGRNFDYHVLHVAE 451

*M.musculus1* LMPNSFQVGSQEYSYEQFLF**NTS**MLVDYGVEALVDAFSRQRAGRIGGGRNFDYHVLHVAV 451

*:*: ..* *: :: :.* :. :*:: ::.:: . ** : * :

*P.monodon* KVIENGREMRFQGMNAYRRRFGMVPFTSFEDLTGETELAAILEEMYGDIEAVEFYVGLLA 536

*P.vannamei* KVIENGRELRFQGVNAYRRRFGMVPFTSFEDMTGETELAAILEEMYGDIEAVEYYVGLLA 535

*M.japonicus* KVLENGRELRFQGVNAYRRRFGMVPFTSFEDMTGETELAAILEELYGDIDAVEYYVGLLA 528

*C.sapidus* KVIENGRLLRFQSLNAYRRRFGMRPFTSFLDLAGDPELAADLEHFYGDIEAVEYYVGLVT 526

*H.americanus* KVIENGRQLRFQGINAYRKRFEMRPFTSFQDLTGDTELAAILEEFYGDIDAVEYYVGLLA 532

*H.rubra* KVIENGRLLRFQSVNNYRRRFGMKPFTSFEDMTGEKEVAAVLEELYGDIEAVEYYIGLLA 538

*Caprella* KMMENGRKLRYQGINEYRKRFALKPFKDFMDLTGDEALAKDLQELYGHVDAVEFYVGLLT 518

*Gammarus* KMLENSRILRFQGVNQYRKKFNMRPFRDFLDLTGDEELARDMEEMYGDINAVEYYVGLIA 518

*O.cuniculus1* EVIKESREMRLQPFNEYRKRFGLKPYASFQELTGETEMAAELEELYGDIDALEFYPGLLL 515

*O.aries1* DVIKESRVLRLQPFNEYRKRFGMKPYTSFQELTGEKEMAAELEELYGDIDALEFYPGLLL 509

*H.sapiens1* DVIRESREMRLQPFNEYRKRFGMKPYTSFQELVGEKEMAAELEELYGDIDALEFYPGLLL 508

*R.norvegicus1* DVIKESREMRLQSFNEYRKRFGLKPYTSFQEFTGEKEMAAELEELYGDIDALEFYPGLML 511

*M.musculus1* DVIKESREMRLQPFNEYRKRFGLKPYTSFQELTGEKEMAAELEELYGDIDALEFYPGLLL 511

.::.:.* :* * .* **::* : *: .* ::.*: :* ::.:**.::*:*:* **:

**S556**

*P.monodon* ERPGP-SVTPLTMVNAGGPWSVKGLLANPICSPRYWKPSTFGGEEGLNIIKTASLEKLFC 595

*P.vannamei* ERPGP-SITPLTMVNIGGPWSVKGLLANPICSPRYWKPSTFGGEEGFNIIKTASLEKLFC 594

*M.japonicus* ERPGP-SITPLTMVNIGGPWSVKGLLANPICSPRYWKPSTFGGEEGFNIIKTASLEKLFC 587

*C.sapidus* ERPGP-SVTPLTMVNMGGPWSVKGLLANPICTPRYWKPSTFGGEEGFQIIKTASLEKLFC 585

*H.americanus* ERPGP-SVTPLSMVNMGGPWSVKGLMANPICSPRYWKPSTFGGEEGFNIIKTASLERLFC 591

*H.rubra* ERPGP-SITPLTMVNIGGPWSVKGLLANPICSPKYWKPSTFGGEVGFNIIQTASVEKLFC 597

*Caprella*  EKDSP-SLTPLTMVNVGGPWSVKGLIANPICSPHWWKPSTFGGDVGFDIVNSASLEKLFC 577

*Gammarus* EKDSP-SLTPLTMVNVGGPWSVKGLIANPICSPHWWKPSTFGGEIGFDIVNTASIEKLFC 577

*O.cuniculus1* EKCQPNSIFGESMIEIGAPFSLKGLLGNPICSPEYWKPSTFGGEVGSNLIKTATLKKLVC 575

*O.aries1* EKCHPNSIFGESMIEMGAPFSLKGLLGNPICSPEYWKASTFGGEVGFNLVKTATLKKLVC 569

*H.sapiens1* EKCHPNSIFGESMIEIGAPFSLKGLLGNPICSPEYWKPSTFGGEVGFNIVKTATLKKLVC 568

*R.norvegicus1* EKCQPNSLFGESMIEMGAPFSLKGLLGNPICSPEYWKPSTFGGDVGFNIVNTASLKKLVC 571

*M.musculus1* EKCQPNSIFGESMIEMGAPFSLKGLLGNPICSPEYWKPSTFGGDVGFNLVNTASLKKLVC 571

*: * *: :*:: *.*:*:***:.****:*.:** *****: * :::::*::::*.*

*P.monodon* LNMKGRCEDIAFTVPQGTP------------- 614

*P.vannamei* LNMKGSCESIGFKVPQGTP------------- 613

*M.japonicus* LNMKGSCEGISFKVPQGTP------------- 606

*C.sapidus* LNMKTGCQNIGFTVPPGTP------------- 604

*H.americanus* LNMNSGCKNIAFSVPAGTP------------- 610

*H.rubra* YNMKSQCQDIAFKVPAGTP------------- 616

*Caprella* NNMKSKCKNIAFKVPRGTP------------- 596

*Gammarus* NNMVSKCQDITFKVPPGTL------------- 596

*O.cuniculus1* LNTK-TCPYVSFRVPRSSGDDGPAAERRSTEL 606

*O.aries1* LNTK-TCPYVSFHVPDPRQEDRPGVERPPTEL 600

*H.sapiens1* LNTK-TCPYVSFRVPDASQDDGPAVERPSTEL 599

*R.norvegicus1* LNTK-TCPYVSFRVPDYPGDDGSVFVRPSTEL 602

*M.musculus1* LNTK-TCPYVSFRVPDYPGDDGSVLVRRSTEL 602

* * : * **
