# Supplementary material for: Biochemical characterization of the cyclooxygenase enzyme in penaeid shrimp
Source: PLoS One. 2021 Apr 22;16(4):e0250276. doi: 10.1371/journal.pone.0250276 (PMC8062024; doi:10.1371/journal.pone.0250276)
Supplement: S2 Fig — (DOCX) [file pone.0250276.s005.docx]

**S2 Fig. Multiple sequence alignment of vertebrate COX2 and invertebrate COXs**

*P.monodon* MSTSVLKTMATTGQRGDKGGGRVMFGVAGVILAASFLFMRTPVHETPAPT--PTAAVIDY 58

*P.vannamei* -MSTSVKSMATTGQRGDKGGGRVVFGVAGVILAASFLFMRTPVHETPAPT--PTAAVIDY 57

*M.japonicus* --------MATTGQRGDKGGGRVMFGVAGVILAASFLFMRTPVHETPAPA--PTEAVIDY 50

*C.sapidus* ---------MDKEGREEKGGRRVMVGVAGVVLAASFLFMRGPVQETPVP---STQPTYDY 48

*H.americanus* ----MVKNMDTEQQQEDKGGGRVLFGVAGVVLAASFLFMRGPAQEAPA--APPSPAPLDY 54

*H.rubra* MSTNEVKSMNTLDWFRDDGDKKLVVGVAGIILAASFLFIRSPVTEPATTVIPQATATYDY 60

*Caprella* --------MQAMSDRRR---AIGFIAV--VAVVWSLSFSGKP-------QNNRVATTLDY 40

*Gammarus* --------MDTMNDRRR---TAVLIVI--AALAWNYLGKEKS-------SSIRIATAVDY 40

*O.cuniculus2* ------------------------------MLARALLL------------CAAVALSHAA 18

*O.aries2* ------------------------------MLARALLL------------CAAVV-CGAA 17

*H.sapiens2* ------------------------------MLARALLL------------CAVLALSHTA 18

*R.norvegicus2* ------------------------------MLFRAVLL------------CAALALSHAA 18

*M.musculus2* ------------------------------MLFRAVLL------------CAALGLSQAA 18

:

**N79 N93**

*P.monodon* DPCCAYPCENQGICMSKPDR**NYT**CDCTGTGHYGTNCEIPTWSTAIKNKLKPDPEWLHTFI 118

*P.vannamei* DPCCAYPCENQGICMSKPDR**NYT**CDCTGTGYYGTNCEIPTWSTAIKTKLKPDPEWLHTFI 117

*M.japonicus* DPCCAYPCENQGICLSEANR**NYT**CDCTGTGYYGSNCEIPTWSTMIKTKLKPDPEWLHTFL 110

*C.sapidus* DPCCSFPCENQGICMTLQNNSYTCDCTGTAHYGSNCEIPTWGSWISKHLKPDPETIHNLI 108

*H.americanus* DPCCAYPCLNLGVCLSFPSN**NYT**CDCTGTDHYGEHCEIPTWRGWLKKTLKPDPETIHNLI 114

*H.rubra* DPCCGYPCLNQGVCMTWPGN**NYT**CDCTGTGHYGTHCEIPTWSAWLVKKIKPDPESLHNFL 120

*Caprella* DPCCGYPCGNQGVCMSFPDRSYECDCTNTGHYGA**NCS**KAEFMTTVVKLIKPDPEYLHILL 100

*Gammarus* DPCCEFPCQNQGVCMSYADKSYSCDCTNTGYYGV**NCT**SATVMTAVINAVKPDPEYLHTIL 100

*O.cuniculus2* NPCCSNPCQNRGVCMTMGFDQYKCDCTRTGFYGE**NCS**TPEFLTRIKLLLKPTPDTVHYIL 78

*O.aries2* NPCCSHPCQNRGVCMSVGFDQYKCDCTRTGFYGE**NCT**TPEFLTRIKLLLKPTPDTVHYIL 77

*H.sapiens2* NPCCSHPCQNRGVCMSVGFDQYKCDCTRTGFYGE**NCS**TPEFLTRIKLFLKPTPNTVHYIL 78

*R.norvegicus2* NPCCSNPCQNRGECMSIGFDQYKCDCTRTGFYGE**NCT**TPEFLTRIKLLLKPTPNTVHYIL 78

*M.musculus2* NPCCSNPCQNRGECMSTGFDQYKCDCTRTGFYGE**NCT**TPEFLTRIKLLLKPTPNTVHYIL 78

:*** ** * * *:: .* **** * .** :* : :** *: :* ::

**N129** **R146 N170**

*P.monodon* TSNGWLWAILNRLPFIHKRLMTYVYLSRGDLVDSPPTYESEHSYITLNAYY**NES**FYGRAL 178

*P.vannamei* TSHSWLWAIINRIPFIHKRLMTYVYLSRGDLVDSPPTYESEHSYITLNAYY**NES**FYARTL 177

*M.japonicus* TSNSWLWAILNRIPFIHKRLMTYVYLSRGDMVDSPPTYESDHSYITLNAYF**NES**YYARTL 170

*C.sapidus* TSCWPLWAVINRIPFLHSRIMTYVYLSRGDLVDSPPTYESDHHFITLDAYY**NES**YYGRAL 168

*H.americanus* TSNSLLWAVLNRISFIHKRLMTYVYTSRGDLVDSPPTYESDHTYITLNAYF**NTS**YYGRAL 174

*H.rubra* TSYGWLWAILNRIPFIHDKLMAYVYLSRGDMVDSPPTFESDHSYITLNAYF**NES**YYARAL 180

*Caprella* TSDLWVWKIINNIGFLQDAAMKYIFLSRGDQVDSPVRFESDHSYITLDAYY**NET**YYARTL 160

*Gammarus* TTDLWIWKVINNIPFIHDAAMKYIFLSRGDQVDSPVRFESDHPYTTLDAYF**NET**YFARTL 160

*O.cuniculus2* THFKGVWNIVNSIPFLRNSIMKYVLTSRSHMIDSPPTYNVHYNYKSWEAFS**NLS**YYTRAL 138

*O.aries2* THFKGVWNIVNKISFLRNMIMRYVLTSRSHLIESPPTYNVHYSYKSWEAFS**NLS**YYTRAL 137

*H.sapiens2* THFKGFWNVVNNIPFLRNAIMSYVLTSRSHLIDSPPTYNADYGYKSWEAFS**NLS**YYTRAL 138

*R.norvegicus2* THFKGVWNIVNNIPFLRNSIMRYVLTSRSHLIDSPPTYNVHYGYKSWEAFS**NLS**YYTRAL 138

*M.musculus2* THFKGVWNIVNNIPFLRSLIMKYVLTSRSYLIDSPPTYNVHYGYKSWEAFS**NLS**YYTRAL 138

* .* ::* : *::. * *: **. ::** :: .: : : :*: * ::: *:*

**H233**

*P.monodon* PPVPDHCPTPMGVKGPKEYPDVDELVKKVFLRREFIPEPHETNVLFQYYAQHFTHQFFRT 238

*P.vannamei* PPVPEHCPTPMGVKGPKEYPDVDELIKKVFLRREFIPEPHDTNVLFQYYAQHFTHQFFRT 237

*M.japonicus* PPVPEHCPTPMGVKGPKEYPDVDELIKKVFMRREFIPEPHDTNVLFQYYAQHFTHQFFRT 230

*C.sapidus* PPVPAHCPTPMGVAGPKEFPDVDELIKKVFMRREFIPDPHNTNVLFQYYAQHFTHQFFRT 228

*H.americanus* PPVPEHCPTPFGVKGHKDYPDVDMLIKKVFLRSKFLPEPHNSNLLFQYYAQHFTHQFFRT 234

*H.rubra* PPVPEHCPTPLGDKGYKDYPDVDELIKKVFMRREFIPEPHGTNVLFQYYAQHFTHQFFRT 240

*Caprella* PPIPEHCPTPMGVKGVKELPNLDLLMKKVFARKEFIPDPHDTNLLFQYYAQHFTHQFFRT 220

*Gammarus* PPVPTHCPTPMGVAGKKELPDLDMLIQKVFVRRQFLPEPHDTNLLFQYYAQHFTHQFFRT 220

O.cuniculus2 PPVADDCPTPMGVKGKKELPDSKDVVEKLLLRRKFIPDPQGTNMMFAFFAQHFTHQFFKT 198

*O.aries2* PPVPDDCPTPMGVKGRKELPDSKEVVKKVLLRRKFIPDPQGTNLMFAFFAQHFTHQFFKT 197

*H.sapiens2* PPVPDDCPTPLGVKGKKQLPDSNEIVEKLLLRRKFIPDPQGSNMMFAFFAQHFTHQFFKT 198

*R.norvegicus2* PPVADDCPTPMGVKGNKELPDSKEVLEKVLLRREFIPDPQGTNMMFAFFAQHFTHQFFKT 198

*M.musculus2* PPVADDCPTPMGVKGNKELPDSKEVLEKVLLRREFIPDPQGSNMMFAFFAQHFTHQFFKT 198

**: .****:* * *: *: . :::*:: * :*:*:*: :*::* ::*********:*

*P.monodon* DYKKGPHLTKGTG-GVDVSNIYGPTEQDRRALRSGVDGKLRTQKINGEDFPPYLKDVPGI 297

*P.vannamei* DYKKGPHLTKGTG-GVDVSNIYGLTEQDRRALRSGVDGKLRTQKINGEDFPPYLKDVPGI 296

*M.japonicus* DYKKGPQLTKGTG-GVDVSNIYGLTEQDRRALRSGVDGKLRTQKINGEDFPPYLKDVPGI 289

*C.sapidus* DYAKGPHLTKGNG-GVDVSNIYGLNEQDRRALRSWENGKLRTQVINGEEFPPYLKDVPAI 287

*H.americanus* DYKKGPHITKGTD-GVDVSNIYGLTEQDRQALRSGVNGKLRTQLINGEEFPPYLKDVPAI 293

*H.rubra* DYKKGPQLTKGTG-GVDVSNIYGLTETDRQALRSRVNGKLRTQVINGEEFPPYLKDVPGY 299

*Caprella* NYTMCPQFTKGNG-GVDVSNIYGLTEQHRRAIRMNSDGKLKYQVINDEHYPPYLRDVQGI 279

*Gammarus* NYTKGPQFTKGNG-GVDVSNIYGLTERQRRALRSNVDGKLKFQIINGEHFPPYLKDVPGI 279

*O.cuniculus2* DLKRGPAFTKGLGHGVDLNHIYGETLDRQHKLRLFKDGKMKYQVIDGEVYPPTVKDTQ-V 257

*O.aries2* DIERGPAFTKGKNHGVDLSHVYGESLERQHNRRLFKDGKMKYQMINGEMYPPTVKDTQ-V 256

*H.sapiens2* DHKRGPAFTNGLGHGVDLNHIYGETLARQRKLRLFKDGKMKYQIIDGEMYPPTVKDTQ-A 257

*R.norvegicus2* DQKRGPGFTRGLGHGVDLNHVYGETLDRQHKLRLFQDGKLKYQVIGGEVYPPTVKDTQ-V 257

*M.musculus2* DHKRGPGFTRGLGHGVDLNHIYGETLDRQHKLRLFKDGKLKYQVIGGEVYPPTVKDTQ-V 257

: * :*.* . ***:.::** . :: * :**:: * *..* :** ::*.

*P.monodon* SMDYPPHIPIPEEGKFALGHPFFALLPGLFAYATIWVREHNRVCDELVKVHPDWDDERIY 357

*P.vannamei* SMDYPPHIPIPEEGKFALGHPFFALLPGLFAYATIWVREHNRVCDELVKVHPDWDDERIY 356

*M.japonicus* SMDYPPHIPIPENGKFALGHPFFALLPGLFAYATIWVREHNRVCDELVKLHPSWDDERIY 349

*C.sapidus* SMDYPPHVPIPETGKFALGHPFFALLPGLFAYSTIWMREHNRVCDELLKIHPHWDDERLY 347

*H.americanus* TMDYPPHIPIPEHSKFALGHPFFALLPGLFAYATIWVREHNRVCDELQKIHTDWDDERLY 353

*H.rubra* SMDYPANVPIPETGKFALGHPFFALLPGLFAFSTIWVREHNRICDELLKVHPDWSDERLY 359

*Caprella* EMDYPPHIPITEDNKFALGHPFFALLPGLFVFSTIWMREHNRVCDVLKNQHPDWDDERLY 339

*Gammarus* SMEYPPHLPITEDNKFALGHPFFALLPGLFVYSTIWMREHNRVCEVLKEQHPHWDDERLY 339

*O.cuniculus2* EMIYPPHIP--AHLQFAVGQEVFGLVPGLMMYATIWLREHNRVCDVLKQEHPEWDDEQLF 315

*O.aries2* EMIYPPHIP--EHLKFAVGQEVFGLVPGLMMYATIWLREHNRVCDVLKQEHPEWGDEQLF 314

*H.sapiens2* EMIYPPQVP--EHLRFAVGQEVFGLVPGLMMYATIWLREHNRVCDVLKQEHPEWGDEQLF 315

*R.norvegicus2* DMIYPPHVP--EHLRFAVGQEVFGLVPGLMMYATIWLREHNRVCDILKQEHPEWDDERLF 315

*M.musculus2* EMIYPPHIP--ENLQFAVGQEVFGLVPGLMMYATIWLREHNRVCDILKQEHPEWGDEQLF 315

* ** ::* :**:*: .*.*:***: ::***:*****:*: * : * *.**:::

**Y383 Y413/H414 H416**

*P.monodon* QTARLIITGEVIKITIEDYVQHLSQYKLKLNFEPELTHGTRFQYHNRIHAEFNHLYHWHP 417

*P.vannamei* QTARLIIIGEVIKITIEDYVQHLSQYNLRLTFEPELTHGTRFQYHNRIHAEFNHLYHWHP 416

*M.japonicus* HTARLIIVGEVIKITIEDYVQHLSQYKLKLTFEPELTHGTRFQYHNRIHAEFNHLYHWHP 409

*C.sapidus* QTARLIITGEVIKITIEDYVQHLSQYRLRLTFEPHLTHGTHFQYHNRIHAEFNHLYHWHP 407

*H.americanus* QTARLIITGEVIKITIEDYVQHLSQYKLRLTFEPELTHGTNFQYHNRIHAEFNHLYHWHP 413

*H.rubra* HTARLIVVGEVIKITIEDYVQHLSQYKLRLTFEPELTHGTRFQYHNRIHAEFNHLYHWHP 419

*Caprella* QTAKLIITGEVIKITIEDYVQHLSQYKVDLKFKPQVVHGTRFQFDNRINAEFNHLYHWHP 399

*Gammarus* HTAKLIITGEVIKITIEDYVQHLSQYKVDLKFKPQVVHGTRFQFHNRINVEFDHLYHWHP 399

*O.cuniculus2* QTSRLILIGETIKIVIEDYVQHLSGYHFKLKFDPELLFNQQFQYQNRIAAEFNTLYHWHP 375

*O.aries2* QTSRLILIGETIKIVIEDYVQHLSGYHFKLKFDPELLFNQQFQYQNRIAAEFNTLYHWHP 374

*H.sapiens2* QTSRLILIGETIKIVIEDYVQHLSGYHFKLKFDPELLFNKQFQYQNRIAAEFNTLYHWHP 375

*R.norvegicus2* QTSRLILIGETIKIVIEDYVQHLSGYHFKLKFDPELLFNQQFQYQNRIASEFNTLYHWHP 375

*M.musculus2* QTSRLILIGETIKIVIEDYVQHLSGYHFKLKFDPELLFNQQFQYQNRIASEFNTLYHWHP 375

:*::**: **.***.********* *.. *.*.*.: .. .**:.*** **: ******

**N424**

*P.monodon* LIPDTL**NVS**GTDYAIMDMAYSTAPVFKHGLDEFIHSMVRSRAGALTS-RNHAHALYPVLK 476

*P.vannamei* LIPDTL**NVS**GTDYAIMDMAYSTAPVFKHGLDEFIHSMVNSRAGALTS-RNHAHALYPVLK 475

*M.japonicus* LIPDSL**NVS**GTDYAIMDMAYSTAPVFKHGLDEFIHAMVNSRAGALTN-RNHAQVLYPVLK 468

*C.sapidus* LIPDGLEVSNTSYALMDMAFSTAPIFKHGLDNFIHAMVNSRAGALTA-RNHAHVLYPVLK 466

*H.americanus* LIPDTVKVNDTEYAIMDMAFSTAPVFKHGLDNFIHAMATNRAGALTT-RNHAHTLYPVLK 472

*H.rubra* LIPDALEVNGTSYAIMDMAYSTAPIFKHGLDEFIHSMVKSRAGALTN-RNHANVLYPILK 478

*Caprella* LIPDGIQVEDKYYSLMDMAFSTKSVFTHGLDKFIESMATSRAGKLSH-SNHPLVTLPVLK 458

Gammarus LIPEGIKVEDSYYSLMDMAFSTKSVFTHGLDAFVKALVTNRAGKLTS-RNHSPVTVPVLK 458

*O.cuniculus2* LLPDTFQIDDQQYNYQQFLY**NNS**ILLEHGLTQFVESFTRQIAGRVAGGRNVPPAVQKVAK 435

*O.aries2* LLPDVFQIDGQEYNYQQFIY**NNS**VLLEHGVTQFVESFTRQIAGRVAGRRNLPAAVEKVSK 434

*H.sapiens2* LLPDTFQIHDQKYNYQQFIY**NNS**ILLEHGITQFVESFTRQIAGRVAGGRNVPPAVQKVSQ 435

*R.norvegicus2* LLPDTFNIEDQEYTFKQFLY**NNS**ILLEHGLAHFVESFTRQIAGRVAGGRNVPIAVQAVAK 435

*M.musculus2* LLPDTFNIEDQEYSFKQFLY**NNS**ILLEHGLTQFVESFTRQIAGRVAGGRNVPIAVQAVAK 435

*:*: .:: . * :: :.. :: **: *:.::. . ** :: * . : :

*P.monodon* KVIENGREMRFQGMNAYRRRFGMVPFTSFEDLTGETELAAILEEMYGDIEAVEFYVGLLA 536

*P.vannamei* KVIENGRELRFQGVNAYRRRFGMVPFTSFEDMTGETELAAILEEMYGDIEAVEYYVGLLA 535

*M.japonicus* KVLENGRELRFQGVNAYRRRFGMVPFTSFEDMTGETELAAILEELYGDIDAVEYYVGLLA 528

*C.sapidus* KVIENGRLLRFQSLNAYRRRFGMRPFTSFLDLAGDPELAADLEHFYGDIEAVEYYVGLVT 526

*H.americanus* KVIENGRQLRFQGINAYRKRFEMRPFTSFQDLTGDTELAAILEEFYGDIDAVEYYVGLLA 532

*H.rubra* KVIENGRLLRFQSVNNYRRRFGMKPFTSFEDMTGEKEVAAVLEELYGDIEAVEYYIGLLA 538

*Caprella* KMMENGRKLRYQGINEYRKRFALKPFKDFMDLTGDEALAKDLQELYGHVDAVEFYVGLLT 518

*Gammarus*  KMLENSRILRFQGVNQYRKKFNMRPFRDFLDLTGDEELARDMEEMYGDINAVEYYVGLIA 518

*O.cuniculus2* ASIDQSRQMKYQSLNEYRKRFLLKPYESFEELTGEKEMAAELEALYGDIDAVELYPALLV 495

*O.aries2* ASLDQSREMKYQSFNEYRKRFLLKPYESFEELTGEKEMAAELEALYGDIDAMELYPALLV 494

*H.sapiens2* ASIDQSRQMKYQSFNEYRKRFMLKPYESFEELTGEKEMSAELEALYGDIDAVELYPALLV 495

*R.norvegicus2* ASIDQSREMKYQSLNEYRKRFSLKPYTSFEELTGEKEMAAELKALYHDIDAMELYPALLV 495

*M.musculus2* ASIDQSREMKYQSLNEYRKRFSLKPYTSFEELTGEKEMAAELKALYSDIDVMELYPALLV 495

:::.* :::*..* **::* : *: .* :::*: :: :: :* .::.:* * .*:.

**S556**

*P.monodon* ERPGPS-VTPLTMVNAGGPWSVKGLLANPICSPRYWKPSTFGGEEGLNIIKTASLEKLFC 595

*P.vannamei* ERPGPS-ITPLTMVNIGGPWSVKGLLANPICSPRYWKPSTFGGEEGFNIIKTASLEKLFC 594

*M.japonicus* ERPGPS-ITPLTMVNIGGPWSVKGLLANPICSPRYWKPSTFGGEEGFNIIKTASLEKLFC 587

*C.sapidus* ERPGPS-VTPLTMVNMGGPWSVKGLLANPICTPRYWKPSTFGGEEGFQIIKTASLEKLFC 585

*H.americanus* ERPGPS-VTPLSMVNMGGPWSVKGLMANPICSPRYWKPSTFGGEEGFNIIKTASLERLFC 591

*H.rubra* ERPGPS-ITPLTMVNIGGPWSVKGLLANPICSPKYWKPSTFGGEVGFNIIQTASVEKLFC 597

*Caprella* EKDSPS-LTPLTMVNVGGPWSVKGLIANPICSPHWWKPSTFGGDVGFDIVNSASLEKLFC 577

*Gammarus* EKDSPS-LTPLTMVNVGGPWSVKGLIANPICSPHWWKPSTFGGEIGFDIVNTASIEKLFC 577

*O.cuniculus2* ERPRPDAIFGESMVEMGAPFSLKGLMGNPICSPNYWKPSTFGGEVGFKIVNTASIQSLIC 555

*O.aries2* EKPAPDAIFGETMVEAGAPFSLKGLMGNPICSPEYWKPSTFGGEVGFKIINTASIQSLIC 554

*H.sapiens2* EKPRPDAIFGETMVEVGAPFSLKGLMGNVICSPAYWKPSTFGGEVGFQIINTASIQSLIC 555

*R.norvegicus2* EKPRPDAIFGETMVELGAPFSLKGLMGNPICSPQYWKPSTFGGEVGFRIINTASIQSLIC 555

*M.musculus2* EKPRPDAIFGETMVELGAPFSLKGLMGNPICSPQYWKPSTFGGEVGFKIINTASIQSLIC 555

*: *. : :**: *.*:*:***:.* **:* :********: *: *:::**:: *:*

*P.monodon* LNMKGRCEDIAFTVPQGTP------------------------------- 614

*P.vannamei* LNMKGSCESIGFKVPQGTP------------------------------- 613

*M.japonicus* LNMKGSCEGISFKVPQGTP------------------------------- 606

*C.sapidus* LNMKTGCQNIGFTVPPGTP------------------------------- 604

*H.americanus* LNMNSGCKNIAFSVPAGTP------------------------------- 610

*H.rubra* YNMKSQCQDIAFKVPAGTP------------------------------- 616

*Caprella* NNMKSKCKNIAFKVPRGTP------------------------------- 596

*Gammarus* NNMVSKCQDITFKVPPGTL------------------------------- 596

*O.cuniculus2* NNVK-GCPFTSFNVPDPQLTKTVTI**NAS**ASHSRLEDINPTVLLKGRSTEL 604

*O.aries2* SNVK-GCPFTSFSVQDAHLTKTVTI**NAS**SSHSGLDDINPTVLLKERSTEL 603

*H.sapiens2* NNVK-GCPFTSFSVPDPELIKTVTI**NAS**SSRSGLDDINPTVLLKERSTEL 604

*R.norvegicus2* NNVK-GCPFASFNVQDPQPTKTATI**NAS**ASHSRLDDINPTVLIKRRSTEL 604

*M.musculus2* NNVK-GCPFTSFNVQDPQPTKTATI**NAS**ASHSRLDDINPTVLIKRRSTEL 604

*: * *.*
