# Supplementary material for: Biochemical characterization of the cyclooxygenase enzyme in penaeid shrimp
Source: PLoS One. 2021 Apr 22;16(4):e0250276. doi: 10.1371/journal.pone.0250276 (PMC8062024; doi:10.1371/journal.pone.0250276)
Supplement: S1 Data — (PDF) [file pone.0250276.s006.pdf]

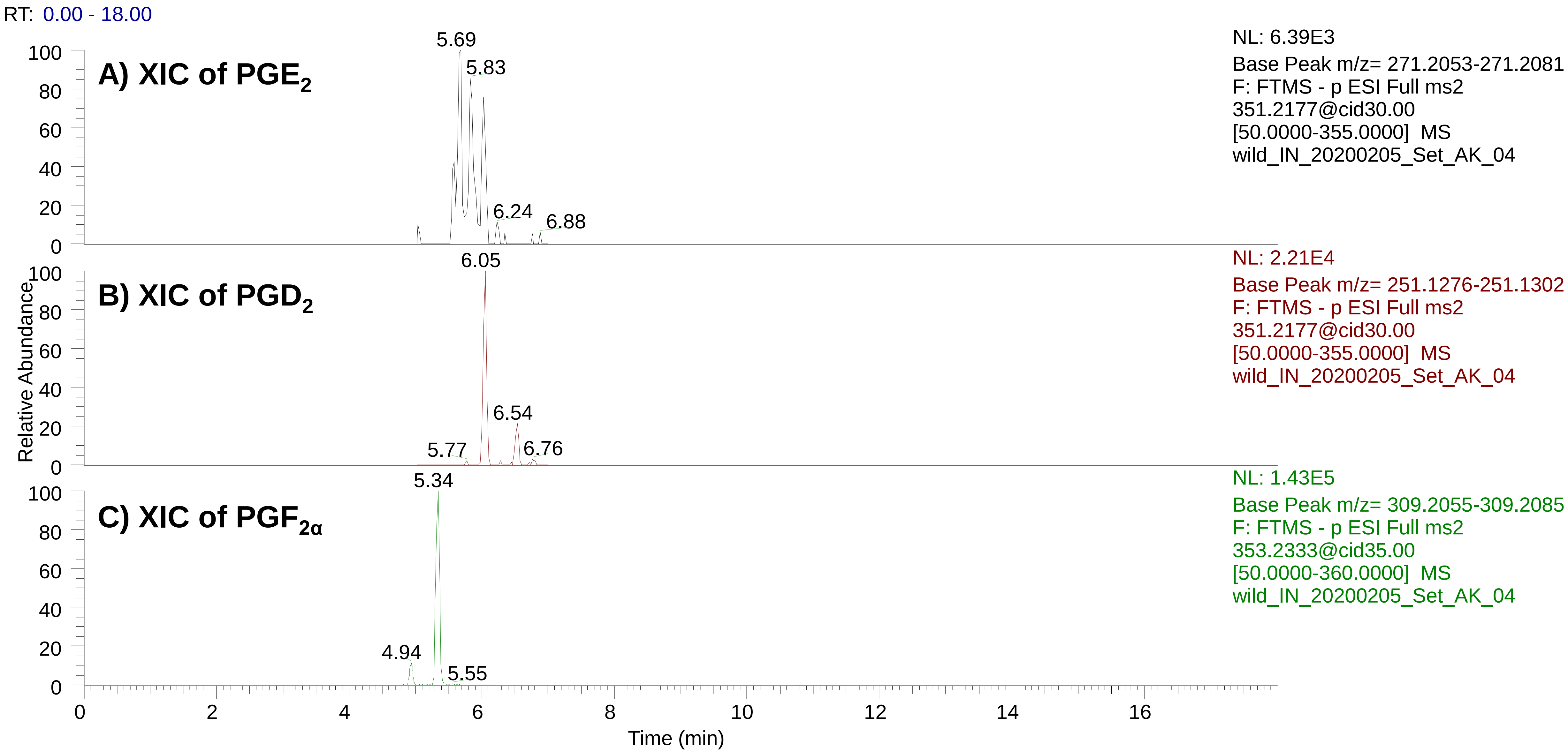

RT: 0.00 - 18.00
A) XIC of PGE2
5.69
5.83
6.24
6.88
NL: 6.39E3
Base Peak m/z= 271.2053-271.2081
F: FTMS - p ESI Full ms2
351.2177@cid30.00
[50.0000-355.0000] MS
wild_IN_20200205_Set_AK_04
B) XIC of PGD2
6.05
5.77
6.54
6.76
NL: 2.21E4
Base Peak m/z= 251.1276-251.1302
F: FTMS - p ESI Full ms2
351.2177@cid30.00
[50.0000-355.0000] MS
wild_IN_20200205_Set_AK_04
C) XIC of PGF2α
5.34
4.94
5.55
NL: 1.43E5
Base Peak m/z= 309.2055-309.2085
F: FTMS - p ESI Full ms2
353.2333@cid35.00
[50.0000-360.0000] MS
wild_IN_20200205_Set_AK_04
Relative Abundance
0
20
40
60
80
100
0
2
4
6
8
10
12
14
16
Time (min)

D) Predicted fragmented ion of PGE_2

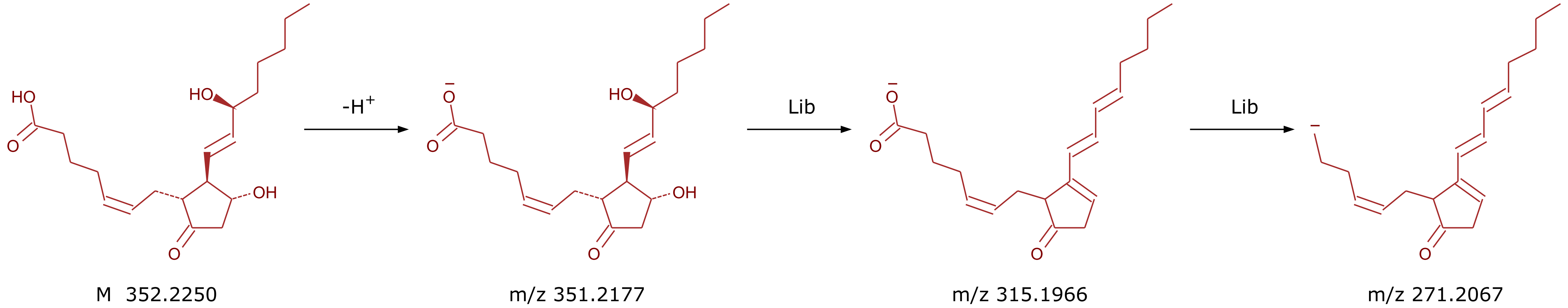

E) Mass spectrum of PGE_2

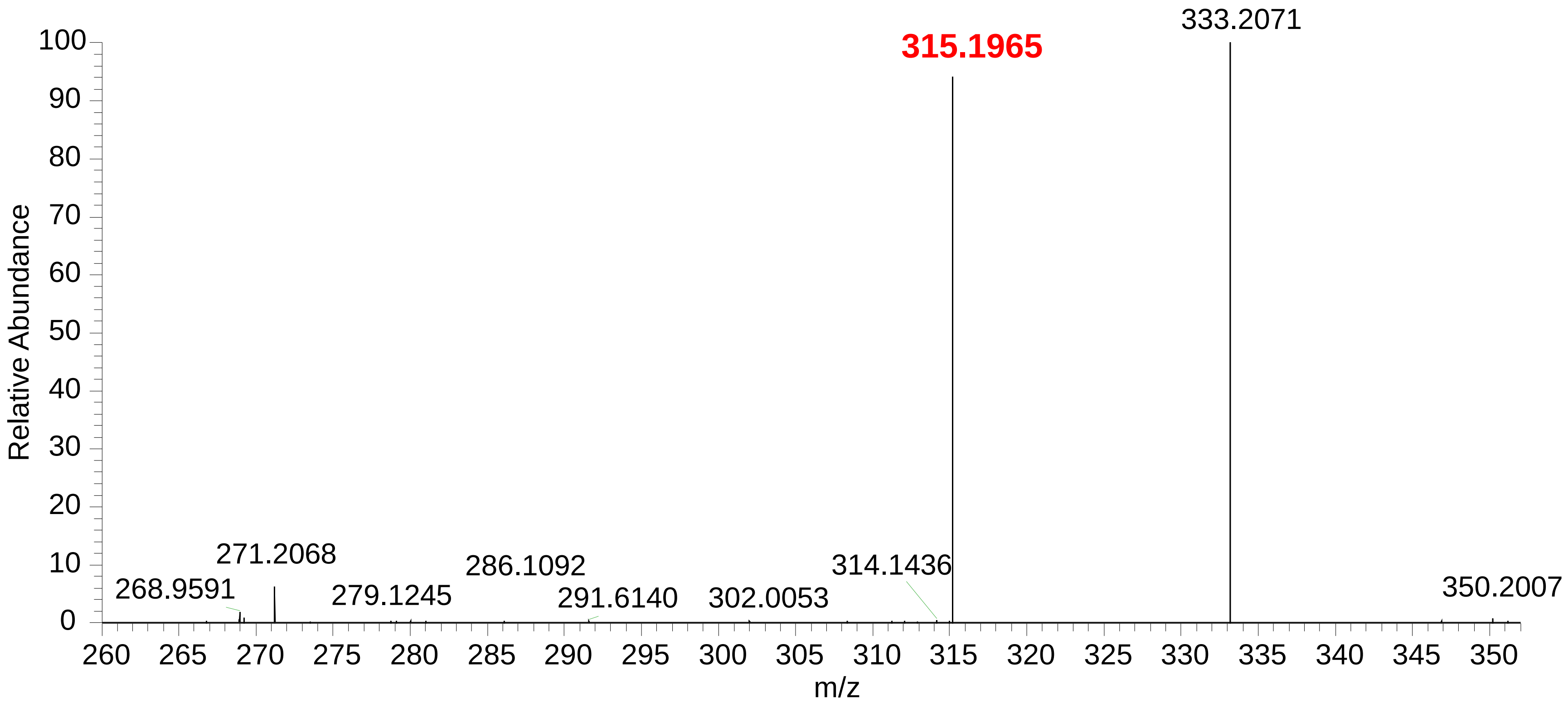

F) Predicted fragmented ion of PGD_2

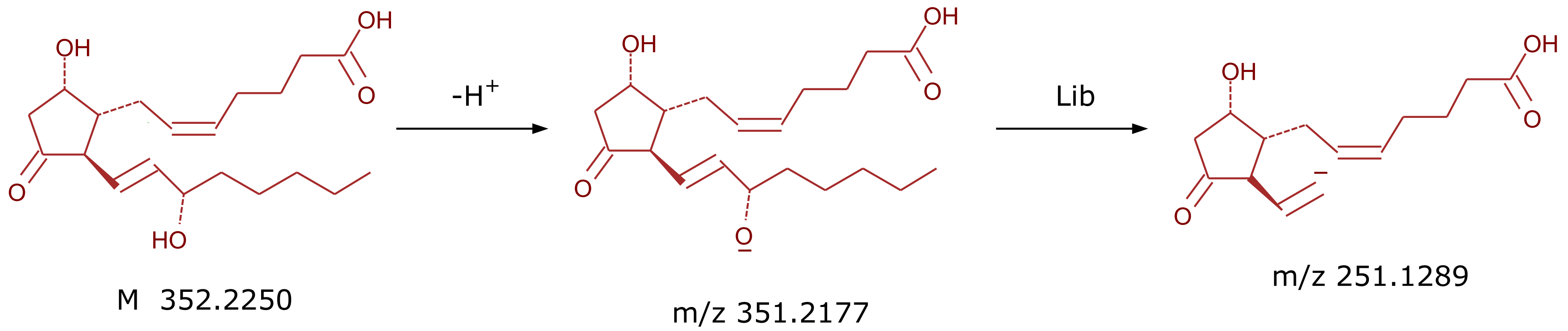

G) Mass spectrum of PGD_2

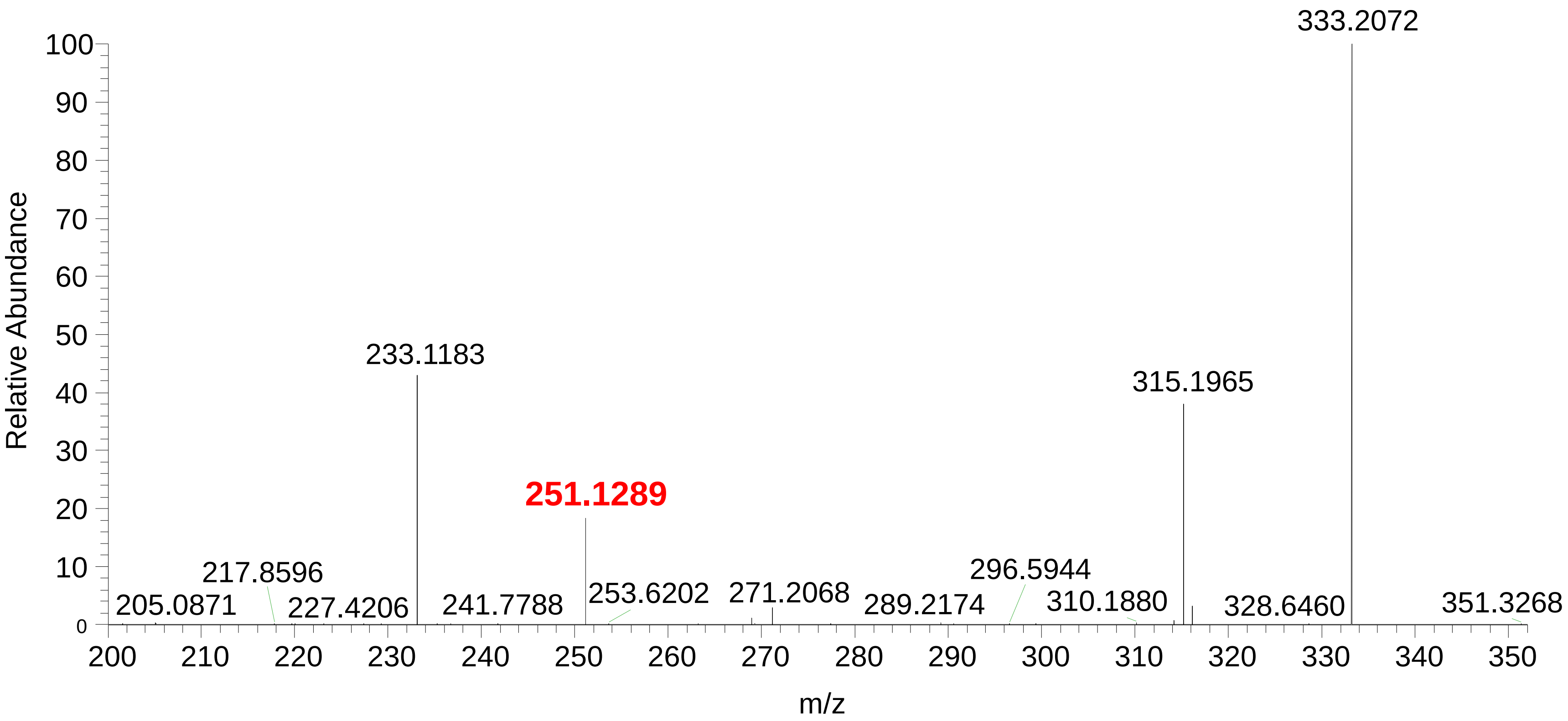

H) Predicted fragmented ion of $PGF_{2\alpha}$

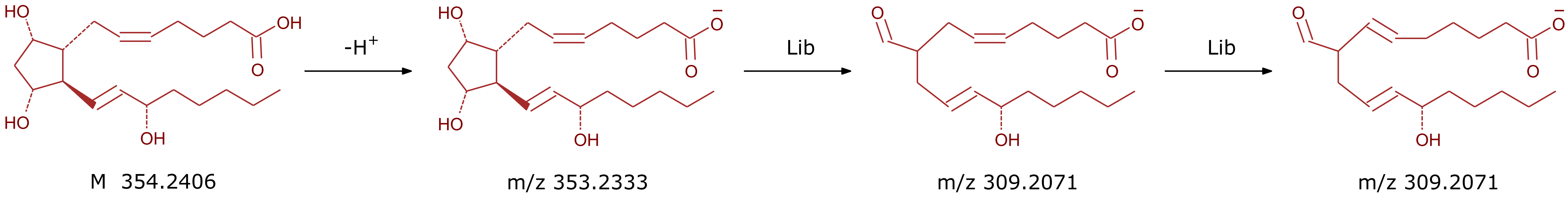

I) Mass spectrum of $PGF_{2\alpha}$

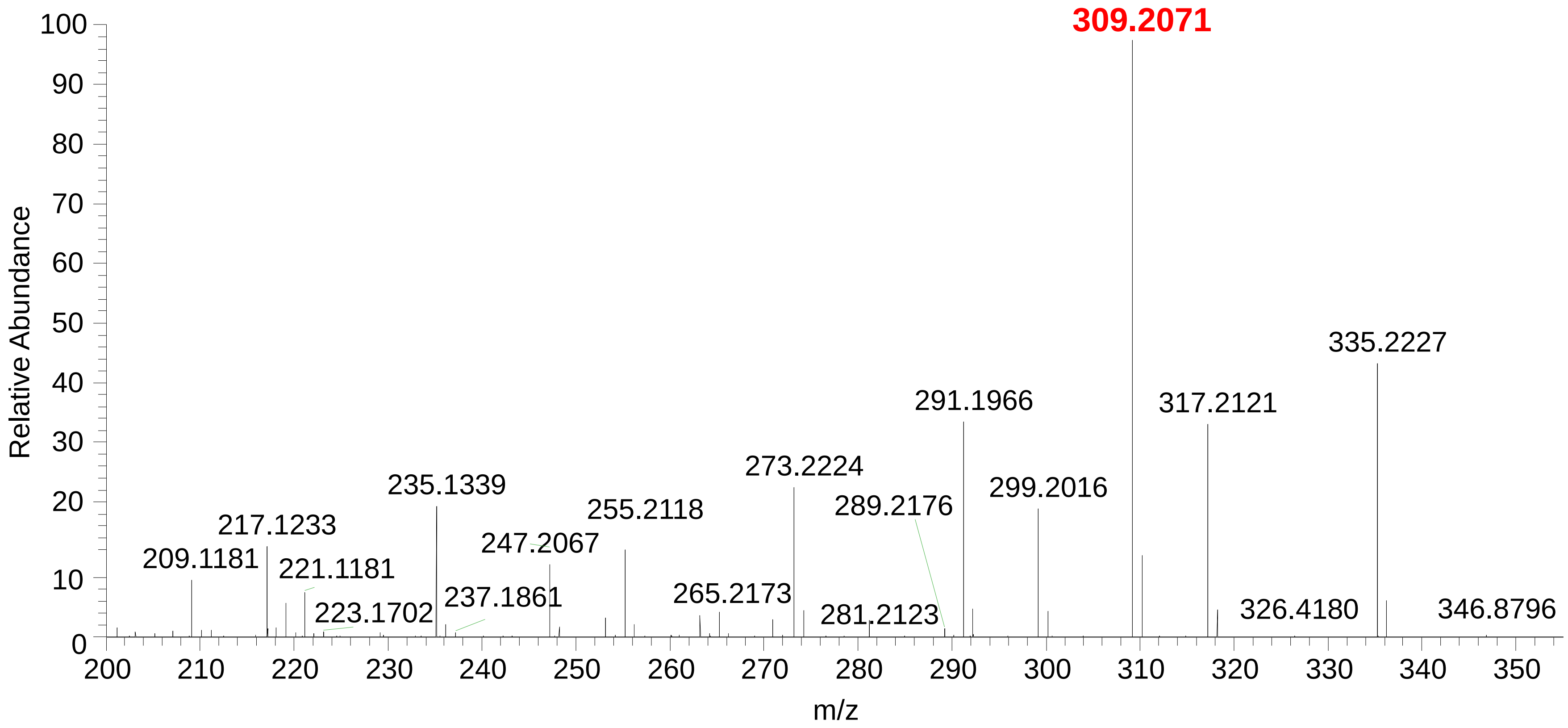

S1 Data: Extracted ion chromatograms and mass spectra of PGE_2, PGD_2 and $PGF_{2\alpha}$ in *P. monodon* intestines. Intestines of wild-caught *P. monodon* broodstock from the Andaman sea were harvested (*n* = 6). Shrimp intestines were homogenized in Hank's balanced salt solution (HBSS) and the tissue concentration was adjusted to 0.1 g/mL. The homogenate was adjusted to pH 4 using acetic acid and subjected to ethyl acetate extraction at a 1:1 ratio (v/v) tissue homogenates:ethyl acetate. The extract was dried using speed vacuum and dissolved in ethanol. UPLC-HRMS/MS analysis revealed extracted ion chromatograms (XIC) of (A) PGE_2, (B) PGD_2 and (C) $PGF_{2\alpha}$. Predicted fragmented ions of (D) PGE_2 (F), PGD_2 and (H) $PGF_{2\alpha}$ matched the mass spectra of (E) PGE_2 (G), PGD_2 and (I) $PGF_{2\alpha}$ obtained from shrimp intestines (red font), respectively.
