# Supplementary material for: Biochemical characterization of the cyclooxygenase enzyme in penaeid shrimp
Source: PLoS One. 2021 Apr 22;16(4):e0250276. doi: 10.1371/journal.pone.0250276 (PMC8062024; doi:10.1371/journal.pone.0250276)
Supplement: S6 Data — (PDF) [file pone.0250276.s011.pdf]

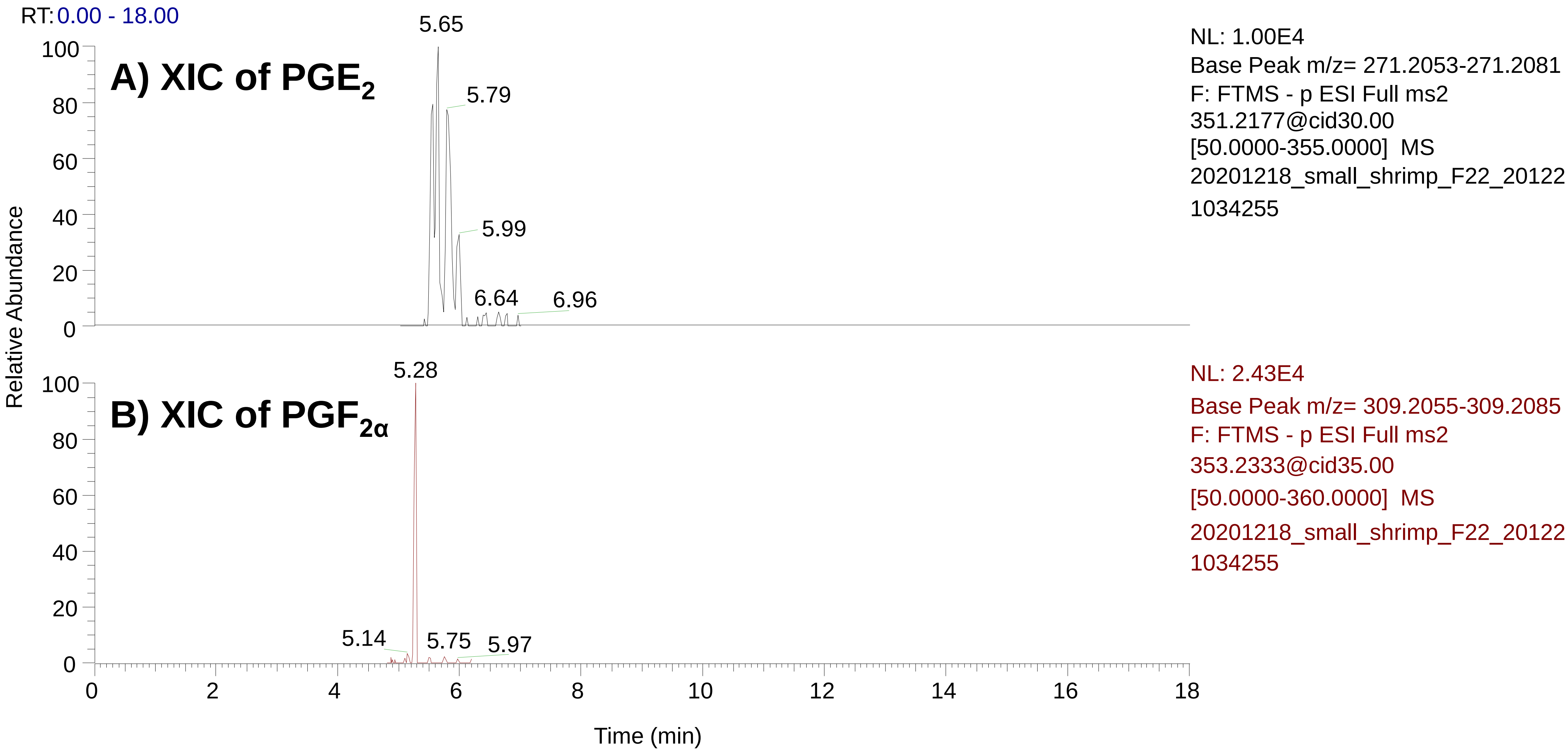

RT: 0.00 - 18.00
A) XIC of PGE2
5.65
5.79
5.99
6.64
6.96
NL: 1.00E4
Base Peak m/z= 271.2053-271.2081
F: FTMS - p ESI Full ms2
351.2177@cid30.00
[50.0000-355.0000] MS
20201218_small_shrimp_F22_20122
1034255
B) XIC of PGF2α
5.28
5.14
5.75
5.97
NL: 2.43E4
Base Peak m/z= 309.2055-309.2085
F: FTMS - p ESI Full ms2
353.2333@cid35.00
[50.0000-360.0000] MS
20201218_small_shrimp_F22_20122
1034255
Relative Abundance
100
80
60
40
20
0
0
2
4
6
8
10
12
14
16
18
Time (min)

C) Mass spectrum of PGE_2

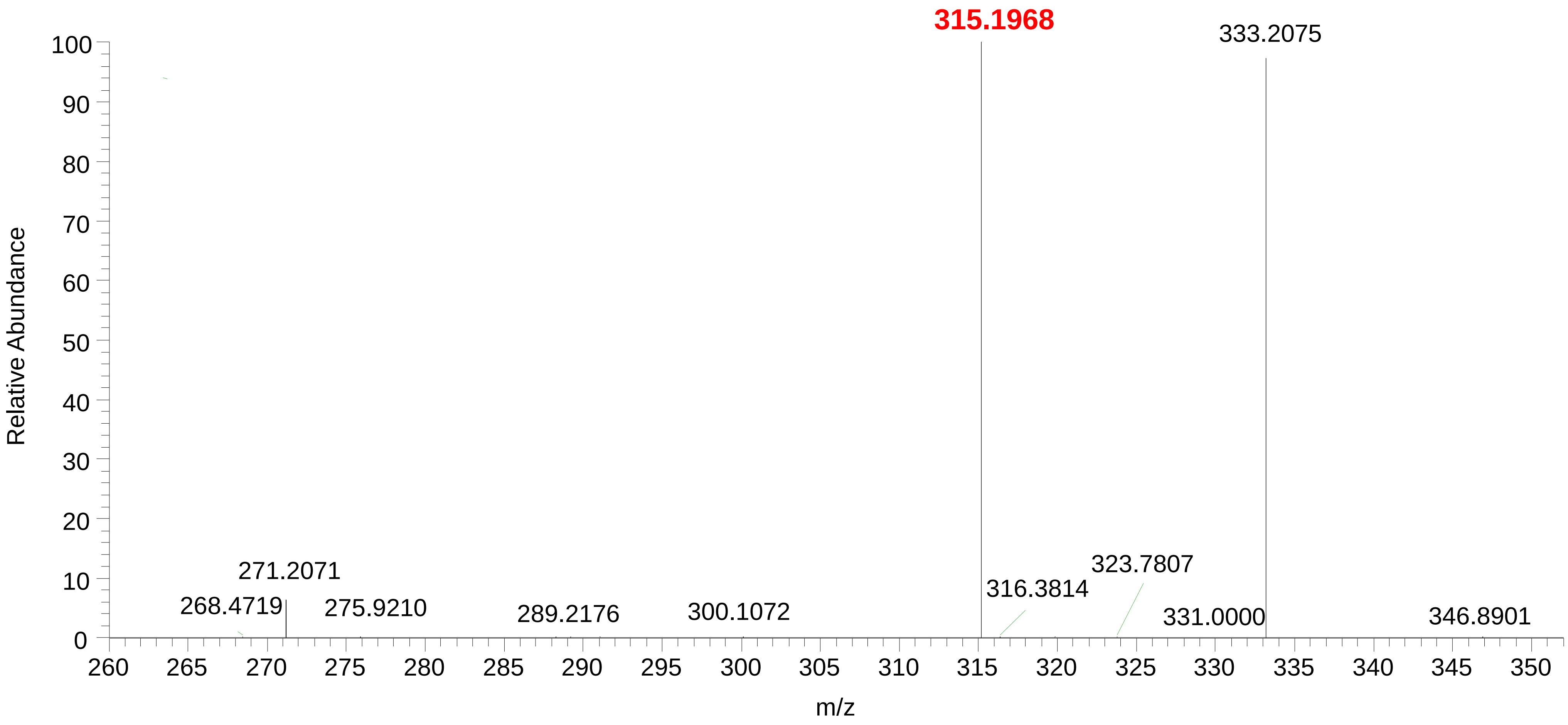

D) Mass spectrum of $PGF_{2\alpha}$

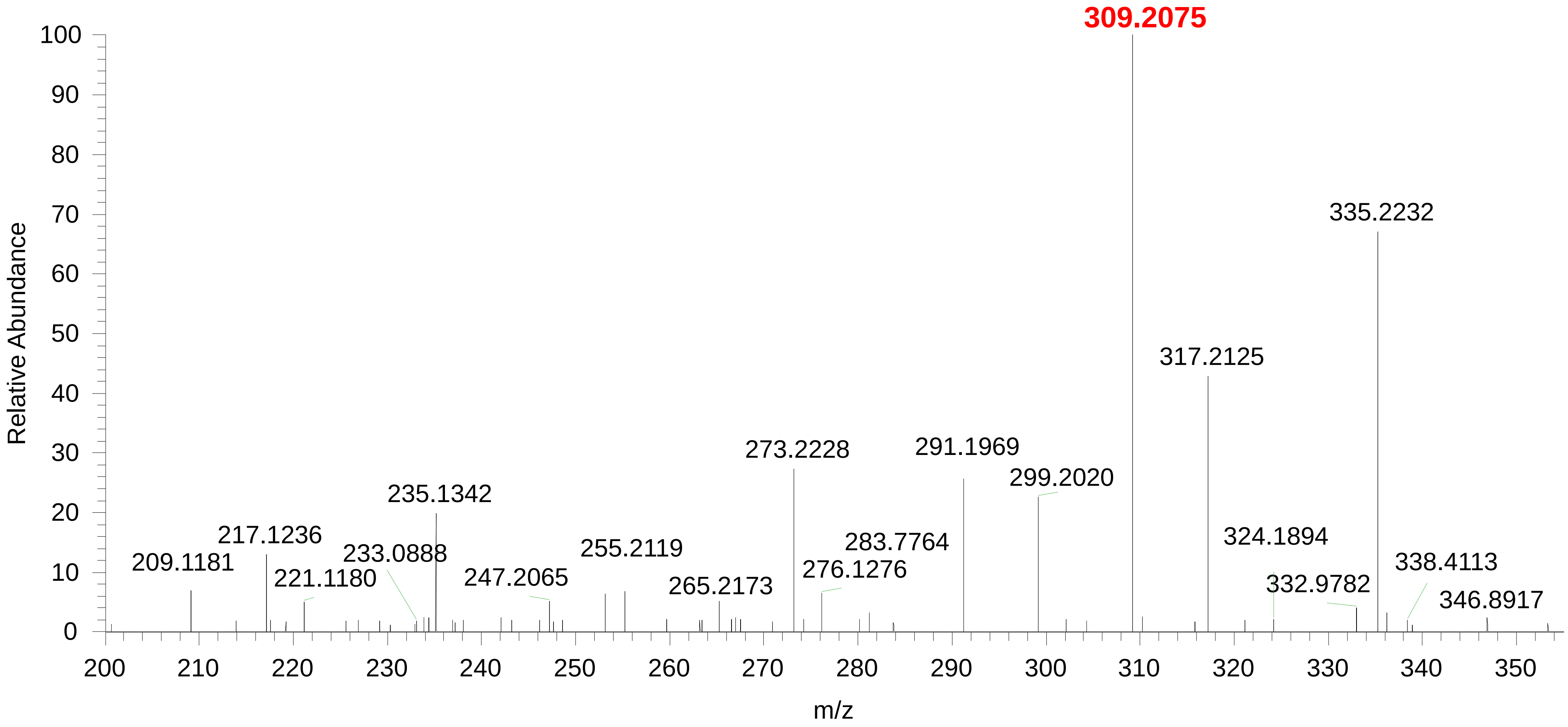

S6 Data. Extracted ion chromatograms and mass spectra of PGE_2 and $PGF_{2\alpha}$ in *P. vannamei* post-larvae.
P. vannamei post-larvae PL18 (*n* = 30) were homogenized in HBSS and the tissue concentration was adjusted to 0.2 g/mL. The homogenate was adjusted to pH 4 using acetic acid and subjected to ethyl acetate extraction at a 1:1 (v/v) ratio of tissue homogenate:ethyl acetate. The extract was dried using speed vacuum and dissolved in ethanol. UPLC-HRMS/MS analysis was performed, revealing XIC of (A) PGE_2 and (B) $PGF_{2\alpha}$. Predicted fragmented ions of PGE_2 and $PGF_{2\alpha}$ matched the mass spectra of (C) PGE_2 and (D) $PGF_{2\alpha}$ obtained from shrimp (red font), respectively.
