# Supplementary material for: Biochemical characterization of the cyclooxygenase enzyme in penaeid shrimp
Source: PLoS One. 2021 Apr 22;16(4):e0250276. doi: 10.1371/journal.pone.0250276 (PMC8062024; doi:10.1371/journal.pone.0250276)
Supplement: S1 Raw images — (PDF) [file pone.0250276.s013.pdf]

Original blot from Fig 3A

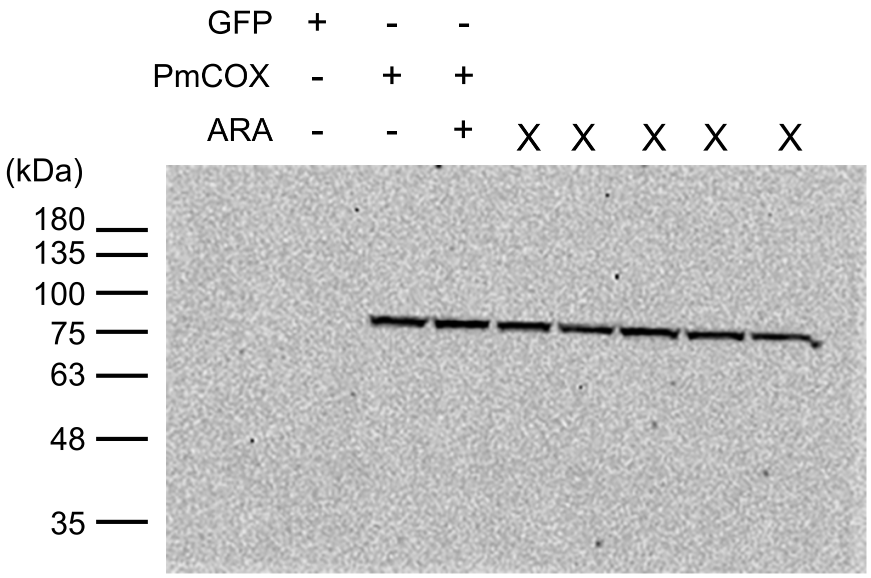

Blot: α-His

293T cells expressing GFP or PmCOX-Myc-His were lysed in RIPA buffer. Western blotting was performed and protein bands were detected using anti-His antibodies. The image was detected using ChemiDoc XRS+ System (BioRad)

X = Sample not included in the manuscript figure
ARA = 293T cells were incubated with arachidonic acids to induce the production of prostaglandins

Original blot from Fig 5A

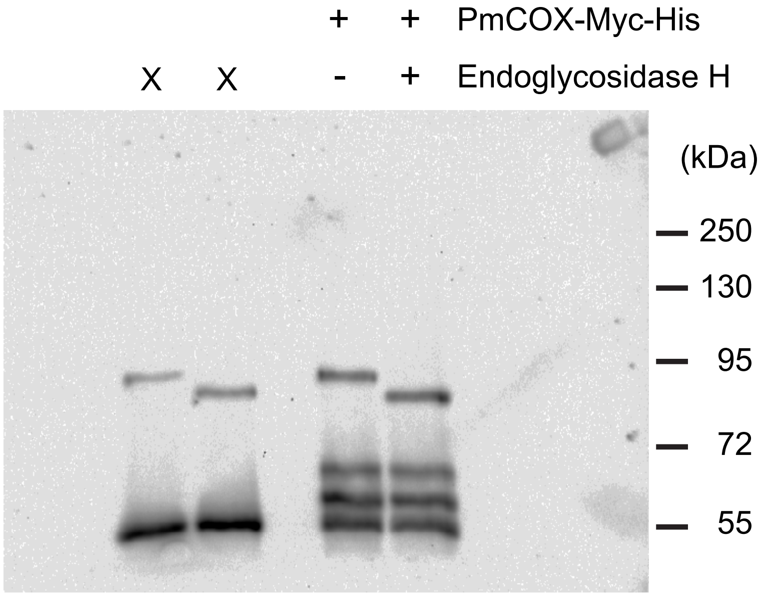

Blot: α-His

293T cells expressing PmCOX-Myc-His protein were subjected to immunoprecipitation using anti-His antibodies. The pulled down proteins were incubated in a reaction mixture in the presence or absence of endoglycosidase H enzyme. The mixtures were then analyzed by Western blotting and probed against anti-His antibodies. The image was detected using ChemiDoc XRS+ System (BioRad)

X = Sample not included in the manuscript figure

Original blot from Fig 5B

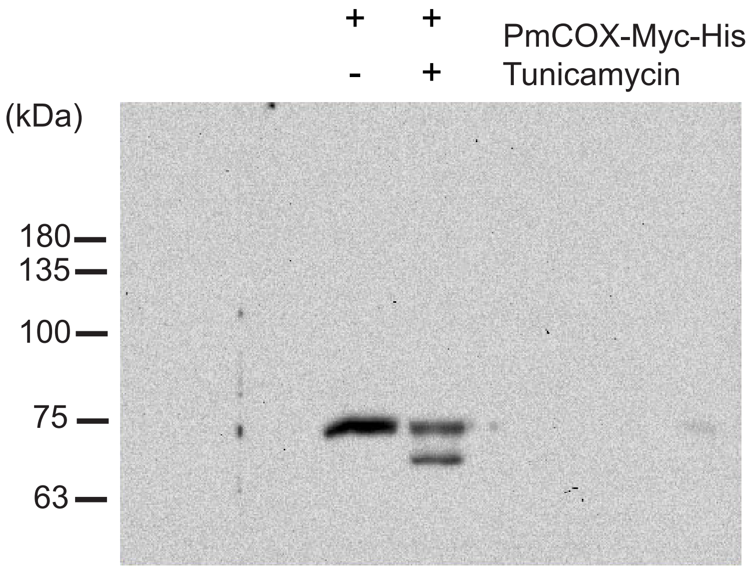

Blot: α-His

293T cells expressing PmCOX-Myc-His were either left untreated or treated with tunicamycin. Western blotting was performed using anti-His antibodies. The image was detected using ChemiDoc XRS+ System (BioRad)

Original blot from Fig 5C

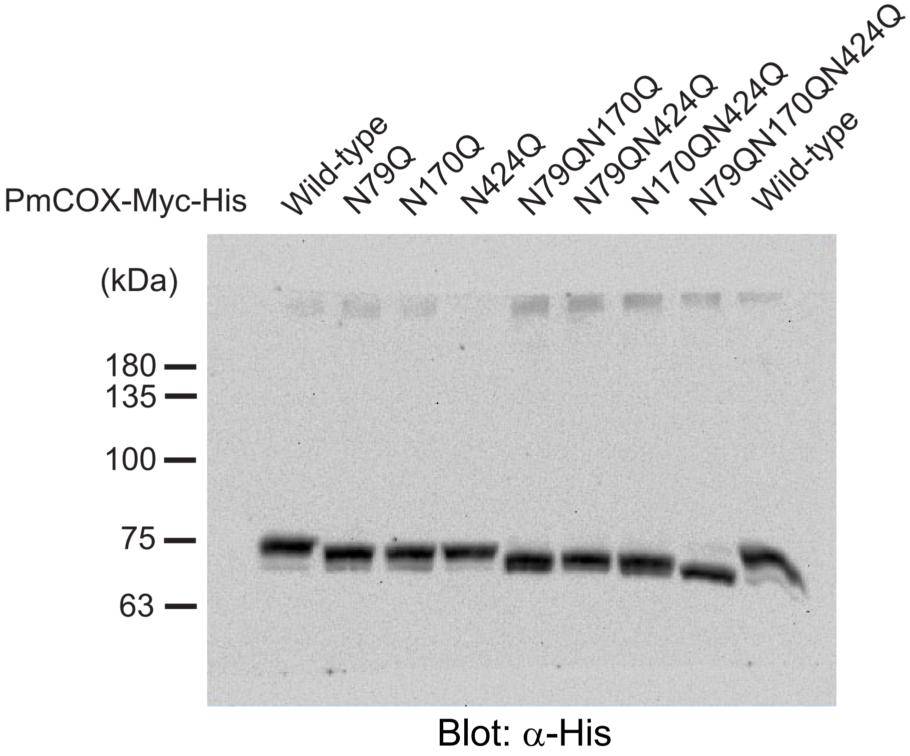

293T cells expressing wild-type PmCOX-Myc-His and PmCOX-Myc-His with single, double, and triple glycosylation mutants. Western blotting was performed and protein bands were detected using anti-His antibodies. The image was detected using ChemiDoc XRS+ System (BioRad)

Original blot from Fig 6A

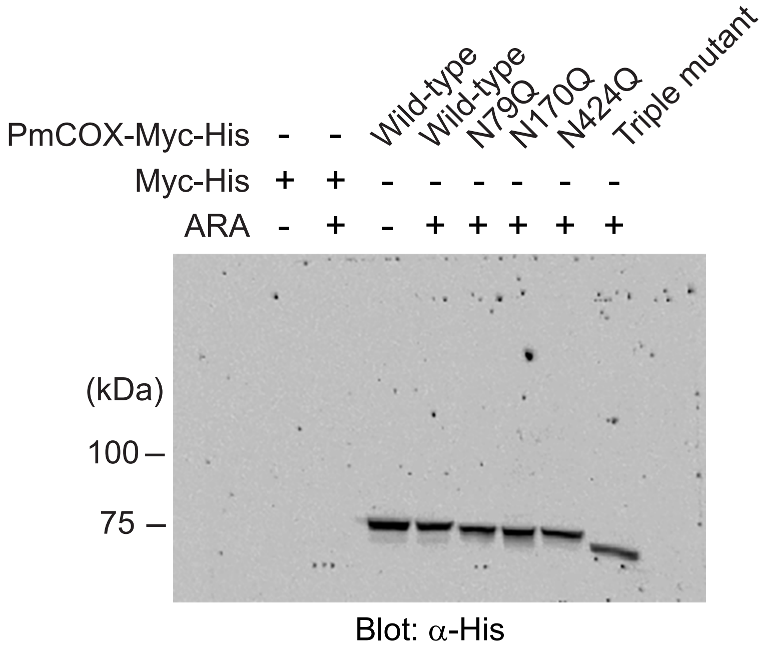

293T cells expressing wild-type PmCOX-Myc-His and PmCOX-Myc-His with single and triple glycosylation mutants. Western blotting was performed and protein bands were detected using anti-His antibodies. The image was detected using ChemiDoc XRS+ System (BioRad)

Myc-His = 293T cells transfected with empty vector
ARA = Cells were treated with arachidonic acid
