# Supplementary material for: Biochemical characterization of the cyclooxygenase enzyme in penaeid shrimp
Source: PLoS One. 2021 Apr 22;16(4):e0250276. doi: 10.1371/journal.pone.0250276 (PMC8062024; doi:10.1371/journal.pone.0250276)
Supplement: Attachment — Submitted filename: Review_PONE-D-20-40833.pdf [file pone.0250276.s014.pdf]

Review: Manuscript ID: PONE-D-20-40833

Tobwar et al. describe the expression and characterization of cyclooxygenase (COX) in penaeid shrimp and identify two asparagine residues that must be glycosylated in the catalytically active enzyme. The authors provide evidence for the enzymatic function both in vitro and in vivo and highlight similarities between various species.

Overall, the work appears to be thorough and well considered. The results are of a high standard and support the conclusions. The paper is well-written and the overall conclusions are placed in the broader context of the potential benefit to the aquaculture industry should this system be leveraged in penaeid shrimp breeding.

Minor issues/suggestions for improvement:

Line	Comment
98	The wording "…analysis performed to obtain the amino acid sequence of PmCOX…" suggests that the sequence was previously unknown. As the sequence is in GenBank, I suggest rewording this to clearly state that sequencing was performed to confirm the correct sequence in the plasmid.
122	Your use of the correct term 'transformation into' (instead of transformation with') is a pleasure to see (and increasingly rare in our field!) – thank you!
155	CV for PGE2 EIA excellent – robust.
160	Comment – pH 8.03 is highly specific; is there a reason why the .03 is noted (is the process that sensitive?)
S1 Table/S1 Data	I am glad to see the standard curves included here and note that the data/figure for PGE_2 is included in S1 Data. For consistency, the standard curve for PGF_2 should also be included in this file.
271/272	Are you able to explain why haemolymph was sampled 48 h post-injection (for potential non-shrimp experts) instead of another timepoint?
294-297	Do the authors have any theories as to why the crustacean COX enzymes lack the C-terminal ER signals?
303	"Predicted glycosylation sites, heme-binding residues, substrate-binding residues and an aspirin acetylation site were identified using multiple sequence alignment". This wording suggests that these sites were predicted using the MSA, but the methods specify the use of NetNGlyc; I suggest rewording to avoid reader confusion. Also, does this mean that the additional residues of interest (heme- and substrate-binding residues and the acetylation site) were identified based on homology with other sequences in the alignment, since no other computational predication tool has been specified?
605	The distal and proximal histidines in PmCOX are identified here but no equivalent residues are mentioned for the other species under discussion here; for consistency with the rest of the paragraph, the equivalent residues should be mentioned along with an explanation of their significance in catalysis (as has been done for R120, Y335 and Y413).
653-4	Comment: It may also be worth investigating whether glycosylation affects dimerization (as in COX2) and whether the role of glycosylation at N79 can be elucidated.
678	This line reads "Our preliminary data revealed that ARA is the preferred substrate for PmCOX, which is supported by the fact that other series of prostaglandins produced from EPA or other precursors have yet to be detected in crustaceans.". While the lack of evidence for other prostaglandins from EPA supports ARA as the preferred substrate (as does the specificity of fish COX1), the work reported here has not actually tested other substrates besides ARA and so, this sentence is misleading. Yes, ARA is a substrate for PmCOX (and yes, it

	is *most likely* preferred), but without the testing of other substrates, it cannot be conclusively stated based on this work said that ARA is preferred. Consider rewording this sentence slightly to avoid overstating this.

Typographical corrections:

**Line**	**Comment**
109/124	Inconsistent capitalization of CLUSTAL OMEGA; please make consistent.
176	Please switch hyphen to an en dash in (-80 degC)
136,<br>143,<br>210, …	Please capitalize ‘western’ in Western blot throughout.
190	Insert space between number and unit (2µM), and capitalize unit.
209	Change hyphen to en dash in -20degC
216	Add space between number and unit (2mM)
617	Typo: Please correct ‘Pichai’ to ‘Pichia’.
